# Influence of mechanical power and its components on mechanical ventilation in SARS-CoV-2

**DOI:** 10.5935/0103-507X.20220018-en

**Published:** 2022

**Authors:** Claudio Luciano Franck, Gustavo Maysonnave Franck

**Affiliations:** 1 Faculdade Evangélica Mackenzie do Paraná - Curitiba (PR), Brazil.; 2 Universidade Tecnológica Federal do Paraná - Curitiba (PR), Brazil.

**Keywords:** Coronavirus infections, SARS-CoV-2, Lung injury, Respiration, artificial, Severe acute respiratory syndrome, Respiratory mechanics

## Abstract

**Objective:**

To analyze the influence of mechanical power and its components on mechanical ventilation for patients infected with SARS-CoV-2; identify the values of the mechanical ventilation components and verify their correlations with each other and with the mechanical power and effects on the result of the Gattinoni-S and Giosa formulas.

**Methods:**

This was an observational, longitudinal, analytical and quantitative study of respirator and mechanical power parameters in patients with SARS-CoV-2.

**Results:**

The mean mechanical power was 26.9J/minute (Gattinoni-S) and 30.3 J/minute (Giosa). The driving pressure was 14.4cmH_2_O, the plateau pressure was 26.5cmH_2_O, the positive end-expiratory pressure was 12.1cmH_2_O, the elastance was 40.6cmH_2_O/L, the tidal volume was 0.36L, and the respiratory rate was 32 breaths/minute. The correlation between the Gattinoni and Giosa formulas was 0.98, with a bias of -3.4J/minute and a difference in the correlation of the resistance pressure of 0.39 (Gattinoni) and 0.24 (Giosa). Among the components, the correlations between elastance and driving pressure (0.88), positive end-expiratory pressure (-0.54) and tidal volume (-0.44) stood out.

**Conclusion:**

In the analysis of mechanical ventilation for patients with SARS-CoV-2, it was found that the correlations of its components with mechanical power influenced its high momentary values and and that the correlations of its components with each other influenced their behavior throughout the study period. Because they have specific effects on the Gatinnoni-S and Giosa formulas, the mechanical ventilation components influenced their calculations and caused divergence in the mechanical power values.

## INTRODUCTION

Severe acute respiratory syndrome 2 (SARS-CoV-2), which causes coronavirus disease 2019 (COVID-19) can generate inflammation and pulmonary fibrosis. Consolidations and bilateral air bronchogram with peripheral distribution of ground-glass opacification in radiological examinations, acute hypoxemia, respiratory failure and a ratio of partial pressure of oxygen/fraction of inspired oxygen (PaO_2_/FiO_2_) less than 300mmHg suggest the diagnosis.^(1)^ Patients infected with SARS-CoV-2 often require high-energy mechanical ventilation (MV) in which the unfavorable prognosis of acute respiratory distress syndrome (ARDS) and ventilator-induced lung injury (VILI) must be weighed.

Acute respiratory distress syndrome and VILI merge into a single and complex form, in which the individual contributions of lung injury are indistinguishable.^(2)^

There is no way to clinically separate the VILI resulting from the underlying process that causes ARDS, but its prediction is made with the progressive reduction of lung volume and unresponsiveness to positive endexpiratory pressure (PEEP) in addition to overcoming the reparative responses^(3)^ that can determine an irreversible acute lung injury. No component of the isolated MV can be held responsible for the VILI, which results in MV configurations and a lung parenchyma condition related to its size, vascular pressure and heterogeneity.^(4-6)^ In addition, VILI depends on the innate vulnerability of the lung tissues.^(7)^ It would be interesting to determine the specific characteristics of VILI that each variable can cause.^(8)^ Mechanical power is a physiological concept that simplifies the evaluation of MV, portraying it as encompassing the set of MV components^(2-9)^ and the injury produced by the energy determined by the pressure gradient, which promotes alveolar deformation.^(10)^ Composed of plateau pressure (P_plateau_), driving pressure (∆P), PEEP, tidal volume (Vt), flow (F), resistance (R), elastance (E_last_) and respiratory rate (RR),^(11,12)^ mechanical power represents the energy transferred from the ventilator to the respiratory system^(13)^ for a period of time in Joules per minute (J/minute).^(9,14)^ The understanding of the biophysical causes of VILI changed the focus of concern of the inflation pattern of the current cycle strictly related to Vt and to the pressures to consider the energy exposures harmful to the lung by mechanical power.^(3)^ The purpose of MV to rest the lungs is compromised when the mechanical strength of its components is inadequately used with increased mechanical power, as the chance of VILI occurring with worsening of the outcome increases.^(9)^ The VILI is influenced by the three stress amplification mechanisms, which are the stress generated by the geometric asymmetry, the accentuation of the forces applied to the microelements by the viscoelastic drag of the flow and the progressive reduction of the microstructures that support the stress, with an increase in the cumulative load in the structures that remain intact.^(15)^ The same load of energy supported without stress or harmful strain in a healthy lung can determine VILI in a “baby lung.”^(3,16)^ The resolution of the injury that determines the alteration of pulmonary mechanics and sufficient tissue repair are necessary or the pulmonary irrecoverability is imminent.^(3)^

This study aimed to analyze the influence of mechanical power and its components on MV in SARS-CoV-2, identify the values of the MV components and verify their relevant correlations with each other and with mechanical power, in addition to their effects on the results of the Gattinoni-S and Giosa formulas.

## METHODS

Longitudinal, observational, analytical and quantitative study of the data collected from the ventilator parameters of patients with SARS-CoV-2 and moderate ARDS admitted to a respiratory intensive care unit of a university hospital, reference in this care modality, from March 2021 to May 2021 in order to analyze the energy provided by the MV and the influence of its components. This study was approved by the Research Ethics Committee of the *Hospital de Clínicas* of the *Universidade Federal do Paraná*, under opinion 4.571.036.

During the study period, 150 patients with SARS-CoV-2 and moderate ARDS who remained intubated in volumecontrolled ventilation with the Puritan Bennet™ 840 respirator and under deep sedoanalgesia and neuromuscular blocker, 510 bits of data were collected from the set of MV components to analyze and insert them into the mechanical power formulas.

The following were considered inclusion criteria for patients: World Health Organization (WHO) severity scale 6 or 7, PaO_2_/FiO_2_ between 100 and 200, chest radiography or tomography with bilateral opacities and D-dimers within the normal range with reverse transcription polymerase chain reaction (RT-PCR) for confirmed SARS-CoV-2.

We chose to collect data from the set of MV components every six days to capture the changes in respiratory mechanics and the behavior of the MV components over the length of time on MV as a result of SARS-CoV-2. The removal of the neuromuscular blocker or death determined the end of the collection.

Respiratory rate, Vt and peak pressure (P_peak_) were recorded, and the P_plateau_ was obtained by inspiratory pause. From these data, the minute volume (Ve), ∆P, resistance pressure (P_resist_), static compliance (C) and E_last_ were generated. The mechanical power was calculated using the formula of Gattinoni et al.^(17)^ simplified (Gattinoni-S) (Equation 1) and the formula of Giosa et al.^(18)^ (Equation 2).


Equation 1 $$\text{ Gattinoni-S }=0,098.FR\cdot Vt\left[P_{\text{peak }}-0,5\left(P_{\text{plateau }}-PEEP\right)\right]$$

Equation 2 $$\text{ Giosa }=0,098\cdot \text{ Ve. }\left(P_{\text{peak }}+PEEP+F/6\right)/20$$

These values and information were transcribed in an Excel® spreadsheet, and statistical analyses of the correlations between the components of MV and mechanical power were performed. Subsequently, the information found in articles published in the literature was discussed.

### Statistical analysis

The quantitative variables were described considering the mean, median, minimum and maximum values, first and third quartiles and standard deviation. The comparison of the Gattinoni-S and Giosa formulas was performed using the Bland-Altman method. To evaluate the association of quantitative variables, the Pearson linear correlation coefficient was estimated. To evaluate the association of the variables of interest with the results of the methods evaluated, simple linear regression and multiple linear regression models were considered. Values of p < 0.05 indicated statistical significance. The *Statistical Package for the Social Sciences*, version 20.0 (IBM Corp., Armonk, New York, United States) and Stata/SE 14.1 (Stata Corp LP, United States) processing systems were used.

## RESULTS

The length of stay of patients included in the study ranged from 8 to 54 days, with a mean of 22 days. During follow-up, of the 150 patients with SARS-CoV-2 and moderate ARDS, 96 died for a mortality rate of 64%.

Among the evaluations of the remaining 510 subjects, the mean ∆P, P_plateau_ and PEEP were 14.4 cmH_2_O, 26.5cmH_2_O and 12.1cmH_2_O, respectively. The mean Vt was 0.36L, the RR was 32 breaths/minute, and E_last_ was 40.6cmH_2_O/L. Table 1 shows the descriptive statistics of the results obtained in the study.

**Table 1:** Results of the mechanical ventilation and mechanical power components

Variables	n	Mean	Minimum	1st quartile	Median	3rd quartile	Maximum	Standard deviation
Flow (L/minute)	510	53.1	24.0	48.0	53.0	58.0	81.0	8.5
Static compliance (L/cmH_2_O)	510	0.028	0.009	0.021	0.027	0.034	0.064	0.009
Vt (L)	510	0.36	0.23	0.32	0.36	0.40	0.60	0.06
Ve (L/minute)	510	11.6	6.0	10.2	11.6	13.0	18.9	2.2
RR (L/minute)	510	32.0	16.0	30.0	33.0	35.0	43.0	3.6
P_peak_ (cmH_2_O)	510	30.6	18.0	27.0	30.0	33.0	47.0	4.7
∆insp (cmH_2_O)	510	18.5	7.0	15.0	17.5	21.0	40.0	5.1
∆P (cmH_2_O)	510	14.4	5.0	11.0	14.0	16.0	34.0	4.6
P_plateau_ (cmH_2_O)	510	26.5	16.0	24.0	26.0	29.0	41.0	4.1
P_resist_ (cmH_2_O)	510	4.1	1.0	3.0	4.0	5.0	13.0	1.9
PEEP (cmH_2_O)	510	12.1	3.0	10.0	12.0	14.0	24.0	3.9
Elastance (cmH_2_O/L)	510	40.6	15.6	29.6	37.5	48.0	109.7	15.2
Resistance (cmH_2_O/L/minute)	510	0.079	0.014	0.055	0.074	0.100	0.243	0.038
Gattinoni-S (J/minute)	510	26.9	9.1	21.2	26.5	31.7	51.9	7.4
Giosa (J/minute)	510	30.3	10.7	24.0	29.6	36.1	58.9	8.3

Vt - tidal volume; Ve - minute volume; RR - respiratory rate; P_peak_ - peak pressure; ∆insp - variation in inspiratory pressure; ∆P - driving pressure; P_plateau_ - plateau pressure; P_resist_ - resistance pressure; PEEP - positive end-expiratory pressure.

The mean mechanical power was 26.9J/minute for the Gattinoni-S formula and 30.3J/minute for the Giosa formula.

**Figure 1:**
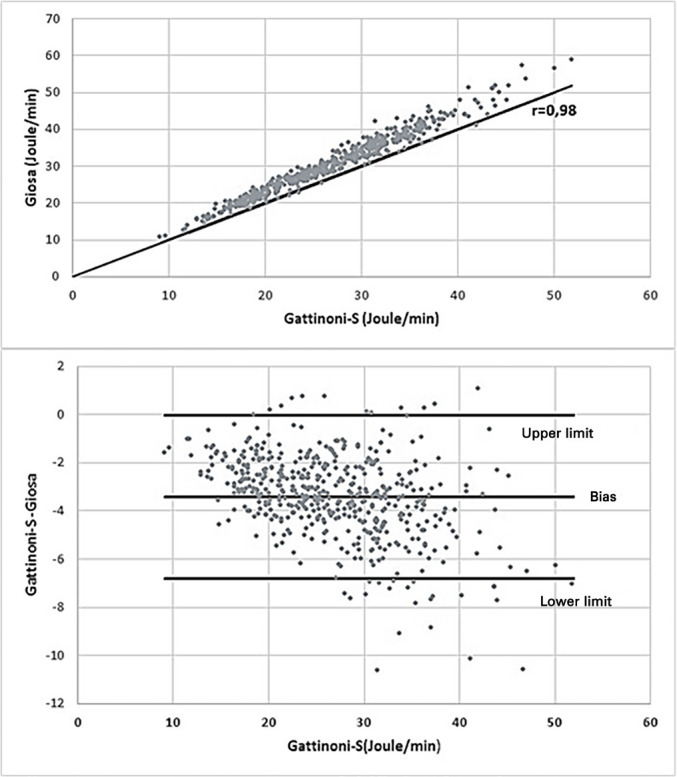
Correlation and bias between Gattinoni-S and Giosa.

To prove the reliability of the results, the hypothesis of the absence of a linear association was tested *versus* the hypothesis of the existence of a linear correlation between the formulas. The bias is shown in the Brand-Altman graph shown in figure 1.

The dispersion diagram shows a strong positive correlation of 0.98 (p < 0.001) between the results of the two formulas, but there was a predominance of higher values with Giosa. The magnitude of the difference between the results depended on the Gattinoni-S value, with an average bias of -3.4J/minute, which was approached when values were low, but as the values increased, the difference between the results of the two formulas increased.

The greatest difference between the results was a value of Giosa 10.62 J/minute higher than the Gattinoni-S value, and in the largest differences, there was a high Vt, between 0.48L and 0.56L, and a low P_resist_, with a maximum of 2cmH_2_O. Ten evaluations showed that the value of Gattinoni-S was higher than the value of Giosa, and in all situations, high P_resist_ was found between 8 and 13cmH_2_O, and in these cases, the largest difference was 1.08J/minute.

To understand the influence of the components on the mechanical power result and the trend of variation of the results between the formulas, the MV components were separately correlated with the results obtained from the formulas, as shown in table 2.

**Table 2:** Correlation between mechanical ventilation components and *mechanical power*

Variables	Gattinoni-S	Giosa
∆P	0.14 (0.001)	0.14 (0.002)
P_plateau_	0.61(< 0.001)	0.58 (< 0.001)
P_resist_	0.39 (< 0.001)	0.24 (< 0.001)
PEEP	0.47 (< 0.001)	0.44 (< 0.001)
Elastance	-0.16 (< 0.001)	-0.20 (< 0.001)
Vt	0.61 (< 0.001)	0.68 (< 0.001)
RR	0.39 (< 0.001)	0.40 (< 0.001)

∆P - driving pressure; P_plateau_ - plateau pressure; P_resist_ - resistance pressure; PEEP - positive end-expiratory pressure; Vt - tidal volume; RR - respiratory rate.

**Figure 2:**
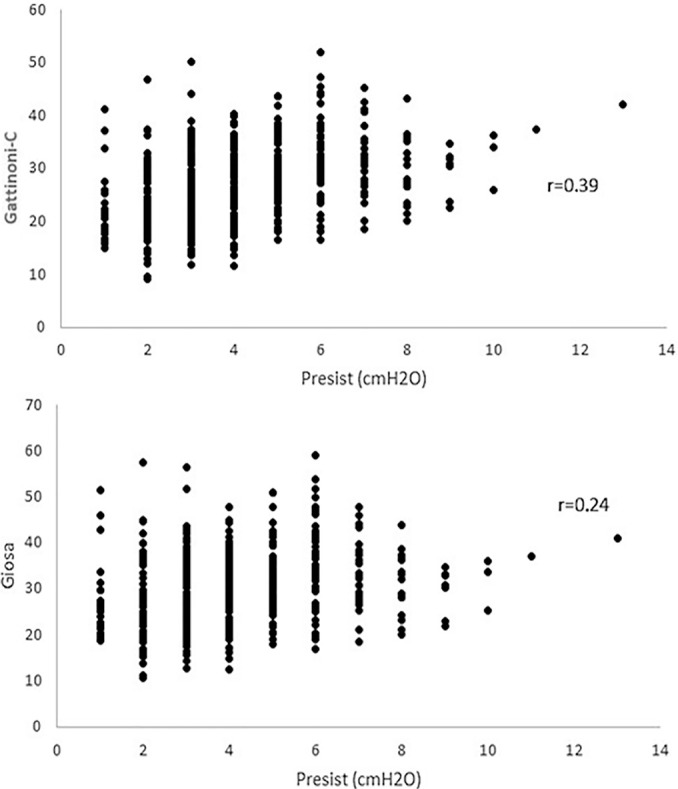
Dispersion diagram between the resistance pressure and formulas P_resist_ - resistance pressure.

The P_plateau_, PEEP, Vt and RR demonstrated a moderate positive correlation, while ∆P had a weak positive correlation. In contrast, the E_last_ was the only one that had a weak negative correlation with both formulas. The only component that showed a significant correlation difference was P_resist_, which had a moderate positive correlation with Gattinoni-S (0.39) and a weak positive correlation with Giosa (0.24), as shown in figure 2.

Table 3 shows the effects of the MV components on the mechanical power values based on the analysis of a multiple regression model. For this analysis, the combined effect of the covariates represented by the P_plateau_, P_resist_, PEEP, E_last_, Vt and RR was evaluated, while the two response variable models were the Gattinoni and Giosa formulas. Due to the strong correlation between ∆P and E_last_ (0.88), these two variables could not be included concomitantly in the models, and for the subsequent analyses, the E_last_ results were considered.

**Table 3:** Effects of the mechanical ventilation components on the formulas

Response variables	Explanatory variables						
	P_plateau_ (cmH_2_O)	P_resist_ (cmH_2_O)	PEEP (cmH_2_O)	Elastance (cmH_2_O/L)	Vt (L)	RR (L/minute)	R^2^ (%)
Gattinoni-S	0.92	1.07	0.20	-0.13	55.79	0.87	96.73
Estimate	0.053	0.023	0.053	0.018	1.993	0.015	
Standard error							
Giosa	0.95	0.60	0.15	-0.14	70.40	1.03	94.54
Estimate	0.078	0.027	0.078	0.026	3.017	0.022	
Standard error							

P_plateau_ - plateau pressure; P_resist_ - resistance pressure; PEEP - positive end-expiratory pressure; Vt - tidal volume; RR - respiratory rate.

If the other components remained with fixed values, for each cmH_2_O increase in the P_plateau_, an increase of 0.92 J/ minute was estimated in Gattinoni-S and 0.95J/minute in the results of Giosa. For each cmH_2_O increase in P_resist_, an increase of 1.07J/minute was estimated in the Gattinoni-S value and 0.60 J/minute in the Giosa results. For each cmH_2_O increase in PEEP, an increase of 0.20J/minute was estimated in Gattinoni-S units and 0.15J/minute in the results for Giosa. Each cmH_2_O/L increase in E_last_ estimated a reduction of 0.13J/minute in the value of Gattinoni-S and 0.14 J/minute of Giosa. For each unit of RR increase, an increase of 0.87J/minute was estimated in the value of Gattinoni-S and 1.03J/minute in Giosa, and for each 0.01L increase in Vt, there was an increase of 0.55J/minute in the Gattinoni-S value and 0.7 J/minute in the results for Giosa.

**Figure 3:**
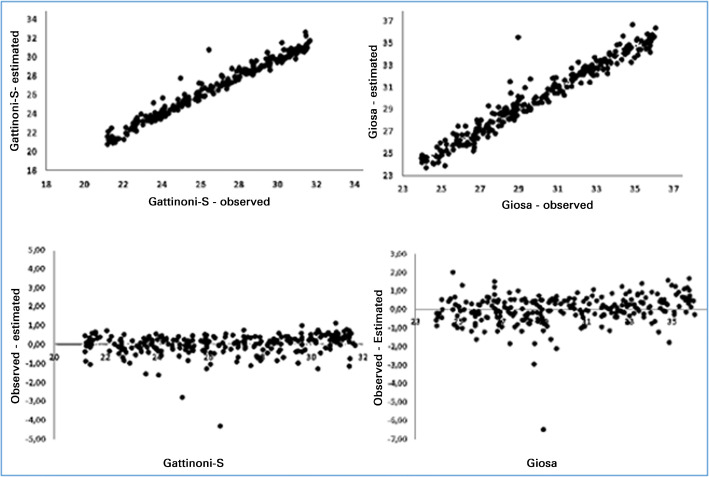
Correlations and differences between the estimated and observed results of the formulas

The models showed good adherence to Gattinoni-S and Giosa values between the first and third quartiles. Figure 3 shows the results of the estimates obtained in the range of 21.7 and 31.7J/minute for Gattinoni-S and 24 and 26.1J/ minute for Giosa.

**Figure 4:**
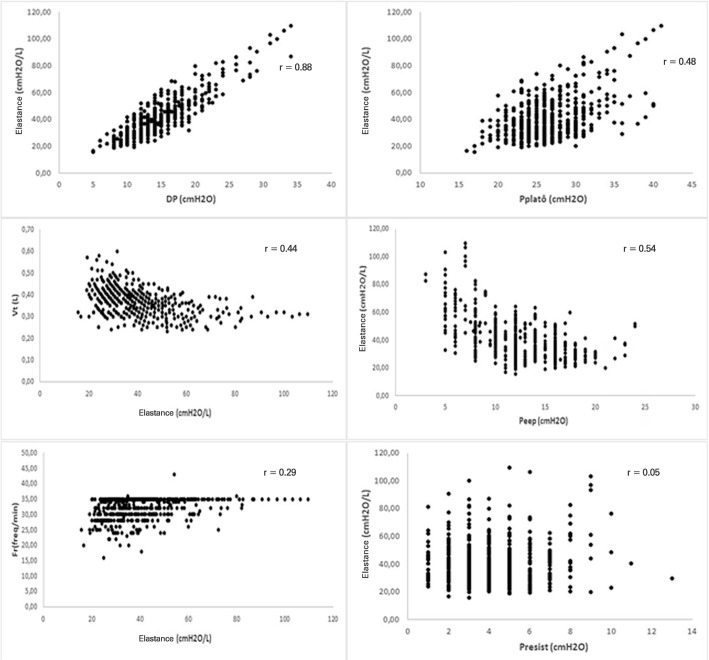
Elastance dispersion diagram with the other components of mechanical ventilation DP - *driving pressure*; P_plateau_ - plateau pressure; Vt - tidal volume; PEEP - positive end-expiratory pressure; P_resist_ - resistance pressure.

In the analysis of these results, greater influence was observed in the results with Vt; when it was high, the value of Giosa, and P_resist_ increased, in turn generating an increase in the value of Gattinoni-S. The largest differences in the value of Giosa compared to Gattinoni-S resulted from high Vt with low P_resist_. Figure 4 shows the behavior of E_last_ and its correlation with the other components of MV. In the intercorrelations of the components with E_last_, there was a strong positive correlation with ∆P (0.88), a moderate positive with P_plateau_ (0.48), a moderate negative with PEEP (-0.54) and with Vt (-0.44), a weak negative with RF and no correlation with P_resist_ (0.05).

## DISCUSSION

The normalization of the mechanical power value as a safety limit for the prevention of VILI depends on lung volume and its distribution in pulmonary heterogeneity.^(12,13)^ The safety thresholds of mechanical power vary for different pulmonary conditions because, in lungs affected by ARDS, the mechanical power values, capable of generating VILI, may be lower than in normal lungs.^(9)^ However, the problem lies in establishing a specific mechanical power value expectation for each triggering factor of pulmonary insult. As normalization is related to the pulmonary condition, which is influenced over time by the evolution of ARDS and VILI, the recognition of these values could help determine the prognosis for patients with SARS-CoV-2.

The original equation allows quantifying the contribution of the components of MV and, in theory, could predict its effects on the lung parenchyma.

However, in clinical practice, the effects of its changes are not always predictable because it is necessary to modify other components to compensate for MV.^(17)^ In this study, in the correlations between the variables, a trend of lower values of Vt and PEEP and higher values of P_plateau_ and ∆P, with the increase of E_last_ were observed.

Complex equations require obtaining ∆P, E_last_ and resistance, while simplified formulas facilitate medical practice with small value bias. The differences in values between the formulas occurred with low and high values due to the influence of the correlations of the components with the formulas and their effects on the results.^(18)^ In this study, there was a tendency of the Giosa values to decline as the Gattinoni-S values increased, which was due to the differences in the effects of the specific components in each formula. In addition, the correlations of the components seem to represent the behavior of the MV components by pulmonary consequences during the course of SARS-COV-2, while the correlations of components with mechanical power seem to represent the need for energy current in relation to the pulmonary condition at a given time.

Divergent variations of mean values and limits of mechanical power were published, such as the mean of 9.1J/minute for normal lungs and 8.8J/minute for ARDS.^(12)^ Another study reported variation in the severity of ARDS between 19 and 24 J/minute.^(19)^ The question remained as to what is the threshold for producing VILI,^(20)^ although it was suggested that mechanical power can cause VILI when it exceeds 12J/minute, and above 17J/minute, it is associated with increased mortality.^(6)^ Even above 13J/ minute, it could cause serious damage.^(21)^ In this study, an average mechanical power equal to 26.9J/minute was found for the Gattinoni-S formula, which is higher than the value of previous studies,^(6,12,19,20,21)^ possibly because the sample is composed only of moderate ARDS generated by SARS-CoV-2.

Differences of up to 2J/minute were considered tolerable in the results of the simplified formulas of mechanical power compared to the geometric method, which is considered the gold standard and obtained by the area generated in the pressure-volume graph of the respirator.^(22)^ In this analysis, the mean value of the results of the two simplified formulas showed a strong positive correlation, but with an average bias of -3.4J/minute and a prevalence of higher values for Giosa. As the Gattinoni-S values increase, the difference between the two formulas becomes more pronounced, a fact that is due to the difference in the specific effects of each MV component on their calculations.

It was shown that the effect of PEEP on the formulas is greater than that of E_last_, and PEEP has a moderate positive correlation, while E_last_ denotes a weak negative correlation with mechanical power. In addition, the correlation of these two components of the MV is moderately negative. For example, the mixture of effects and intricate intercorrelations may result in a higher mechanical power when the lung responds with recruitment to PEEP than in the lung condition that indicates fibrosis of low response to PEEP recruitment and high E_last_. This is due to the greater effect of PEEP on the calculation of mechanical power in relation to E_last_ and the tendency of the correlation between these components, which denotes a reduction in PEEP when E_last_ increases. The high mechanical power, regardless of the combination of its components, can cause VILI.^(11)^ However, pointing to a single MV component with or without the determinant of VILI may be misleading, because it seems more reasonable that VILI is the result of a combination of the various MV components^(8)^ and the interdependence between mechanical power and its components.^(23)^ The question remains as to which of these factors most influences the advent of VILI.^(7)^ It is indicated that isolated mechanical power values cannot provide definitive information on the prognosis of lung recovery capacity because they may represent the severity of ARDS but not that of VILI. Combining these analyses, it is assumed that an interrelation between the values of some MV components and mechanical power could expand the safety margin for VILI diagnosis. A mechanical power value greater than 17J/minute is considered a marker of increased mortality due to VILI,^(6)^ and its worsening may be predicted by nonresponsiveness to PEEP and prone, with progressive reduction of lung volume, in addition to overcoming the opposite reactive responses, which culminate in irreversible acute lung injury.^(3)^ In this study, mean values of mechanical power were found above this level, with a mortality rate of 64%, but it is not possible to state which deaths were determined by VILI or for other reasons.

## CONCLUSION

In the analysis of mechanical ventilation for patients with SARS-CoV-2 infections, it was found that the correlations of its components with mechanical power influenced its high momentary values and that the correlations of its components with each other influenced their behavior throughout the study period. Because they have specific effects on the Gatinnoni-S and Giosa formulas, the mechanical ventilation components influenced their calculations and caused divergence in the mechanical power values.
